# Supplementary figures and images for: EhRacM differentially regulates macropinocytosis and motility in the enteric protozoan parasite Entamoeba histolytica
Source: PLoS Pathog. 2024 Nov 13;20(11):e1012364. doi: 10.1371/journal.ppat.1012364 (PMC11560011; doi:10.1371/journal.ppat.1012364)

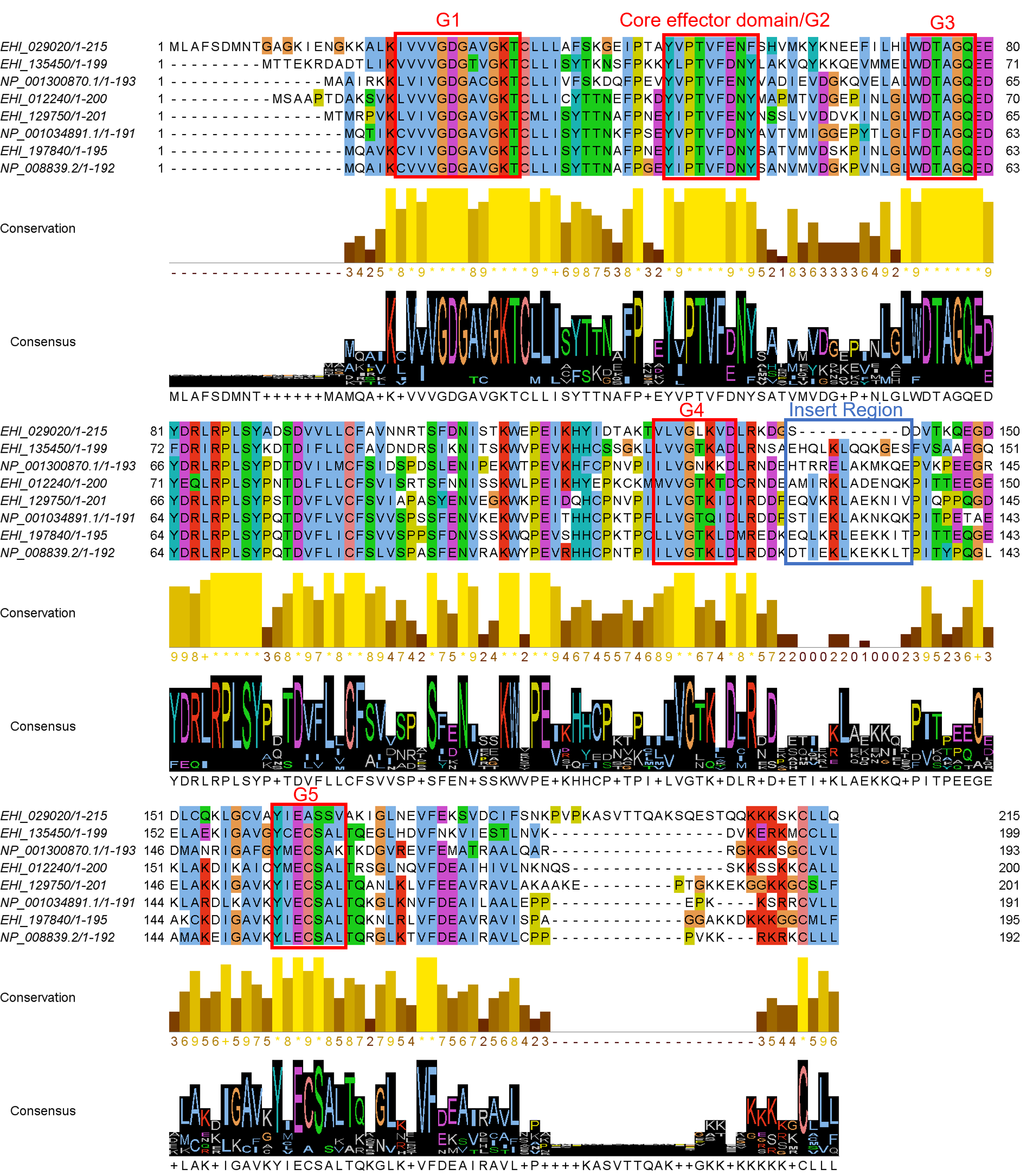

Supplement: S1 Fig — The amino acid sequences of HsRhoA (NP_001300870.1), HsRac1 (NP_008839.2), HsCdc42 (NP_001034891.1), EhRho1B (EHI_029020), EhRacA2 (EHI_197840), EhRacD1 (EHI_012240), EhRacG (EHI_129750), and EhRacM (EHI_135450) were aligned. The conserved G boxes are highlighted in red, while the Rho insert regions are highlighted in blue. (TIF) [file ppat.1012364.s001.tif]

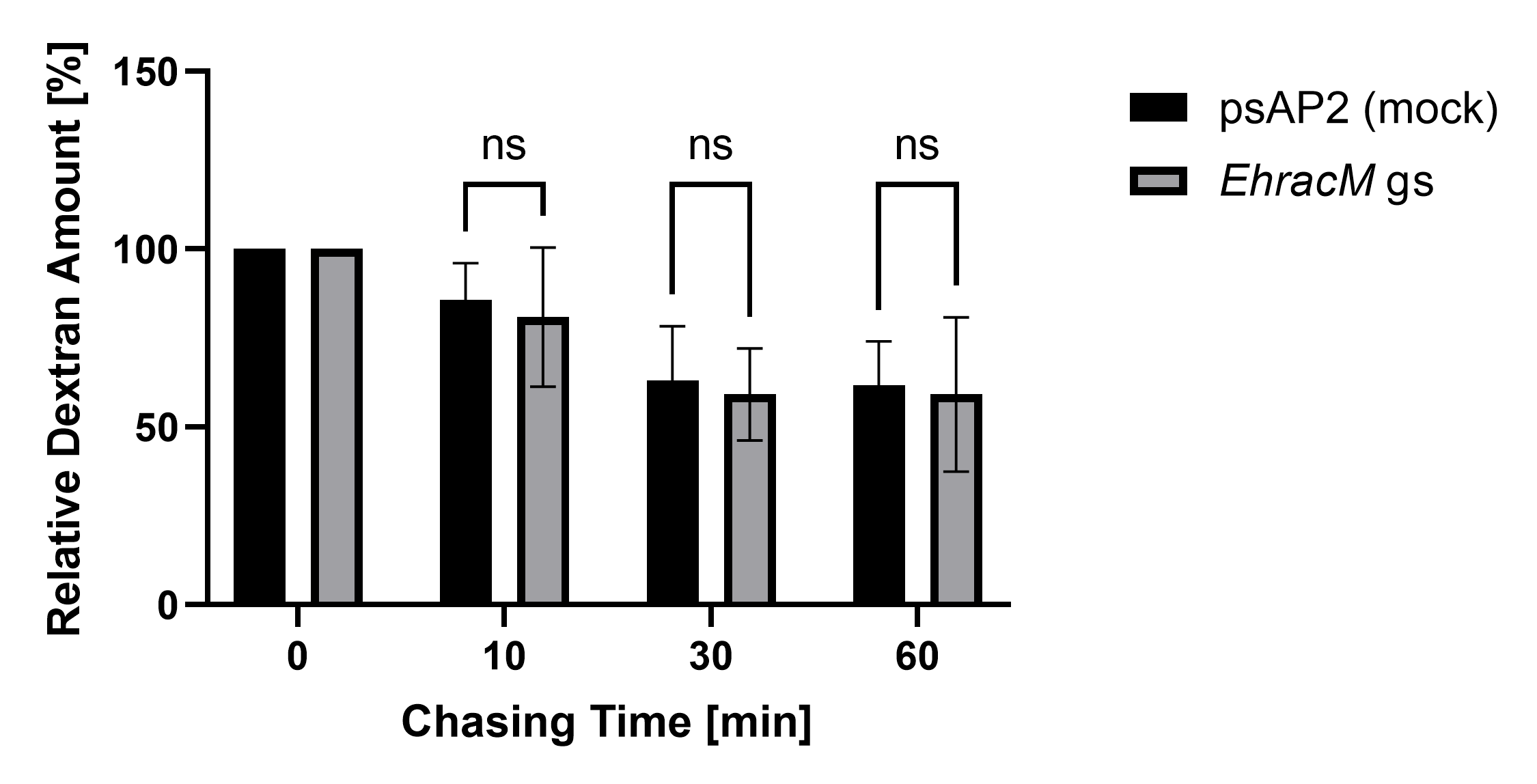

Supplement: S2 Fig — Trophozoites of psAP2 mock and EhracM gene silenced (gs) strains were incubated in BIS medium containing RITC dextran containing for 15 minutes. Amoeba cells were then incubated in dextran-free BIS medium and chased for 0, 10, 30, and 60 minutes. The fluorescence intensity of each amoeba cell was measured by FACS as described in Materials and Methods. The fluorescence intensity relative to the original intensity (time point 0 min, 100%) in each trial was averaged. Statistical significance was examined with an unpaired t-test (ns: not significant). Error bars indicate standard deviations of three biological replicates. (TIF) [file ppat.1012364.s002.tif]

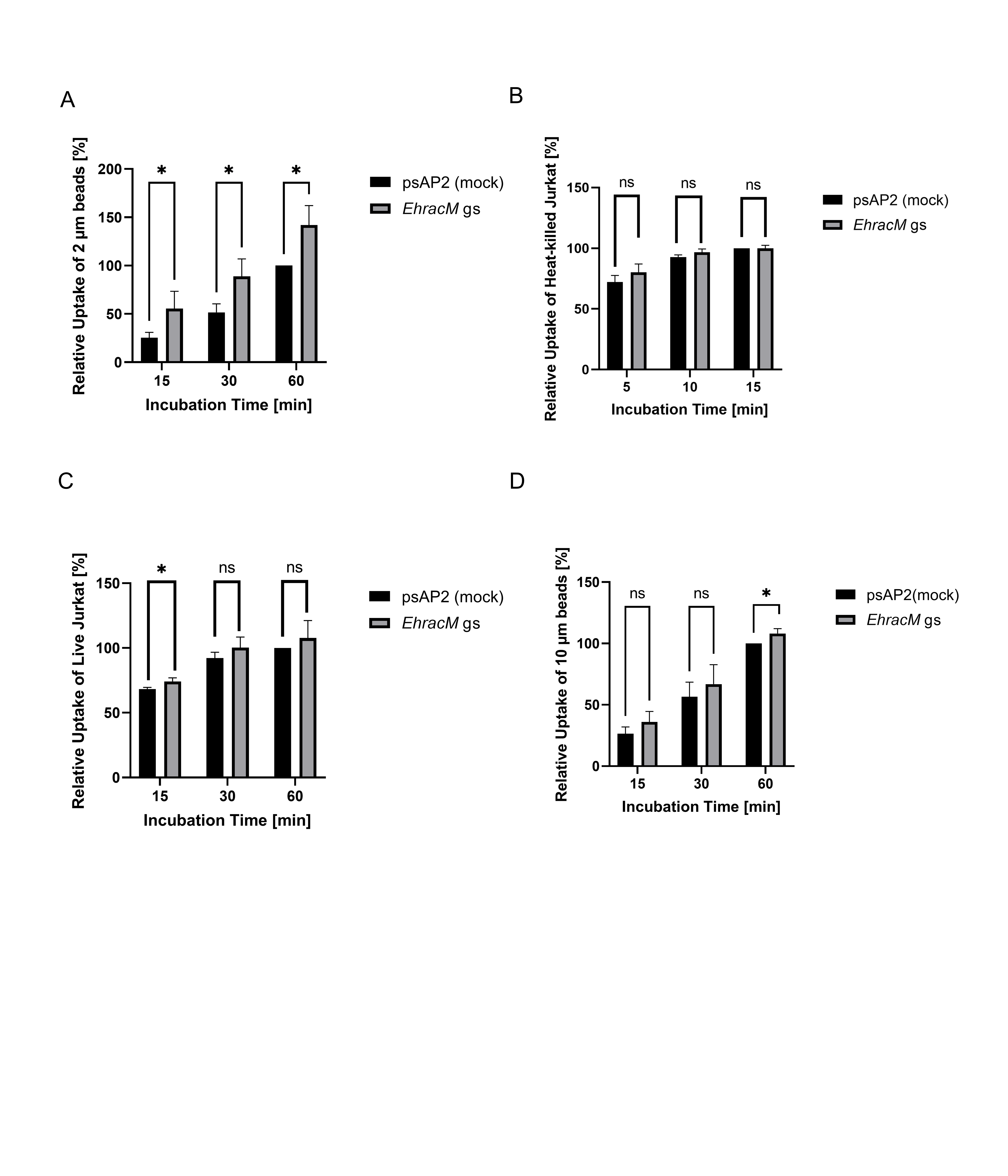

Supplement: S3 Fig — (A), (D) Trophozoites of psAP2 mock and EhracM gene silenced (gs) strains were incubated with 2-μm (A) or 10-μm (D) carboxylated polystyrene beads. The fluorescence intensity of each amoeba cell was measured by FACS as described in Materials and Methods. The relative percentages of amoeba cells containing bead(s) are shown. Each value is standardized by the value of psAP2 mock control strain at 60 min. (B) Trophozoites of psAP2 mock and EhracM gs strains were incubated with pre-killed Jurkat cells, which were heated at 55°C for 15 min. The fluorescence intensity of each amoeba cell was measured by FACS as described in Materials and Methods. The relative percentages of amoeba cells containing Jurkat cell(s) are shown. Each value is standardized by the value of psAP2 mock control strain at 15 min. (C) Trophozoites of psAP2 mock and EhracM gs strains were incubated with live Jurkat cells. The fluorescence intensity of each amoeba cell was measured by FACS as described in Materials and Methods. The relative increase of geometric mean in PE-A channel is shown. Each value is standardized by the value of psAP2 mock control strain at 60 min. Statistical significance was examined with unpaired t-test (*p<0.05, ns: not significant). Error bars indicate standard deviations of three biological replicates. (TIF) [file ppat.1012364.s003.tif]

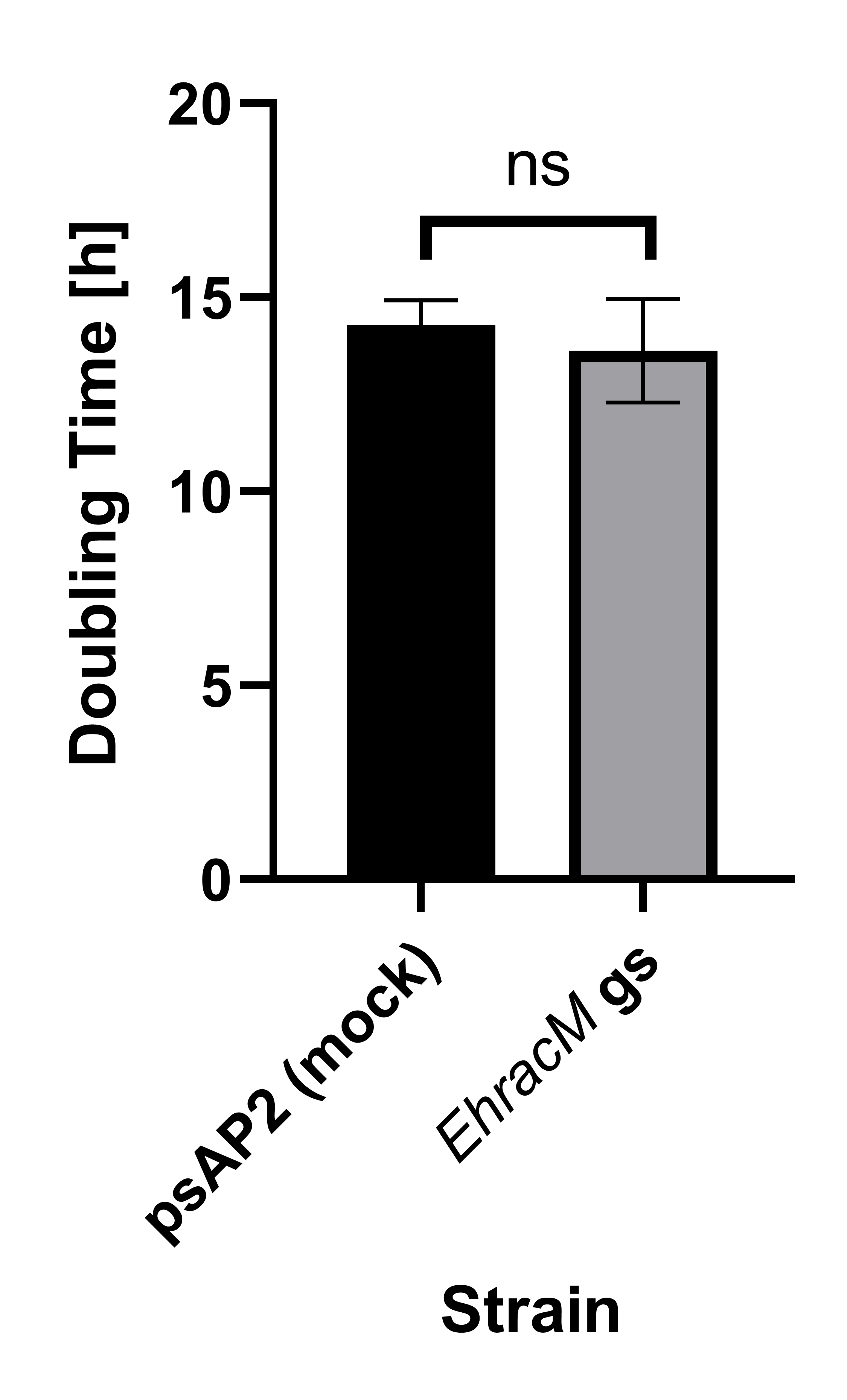

Supplement: S4 Fig — Doubling time of the psAP2 mock and Ehrac gene silenced (gs) strains. Statistical significance was examined with unpaired t-test (ns: not significant). Error bars indicate standard deviations of three biological replicates. (TIF) [file ppat.1012364.s004.tif]

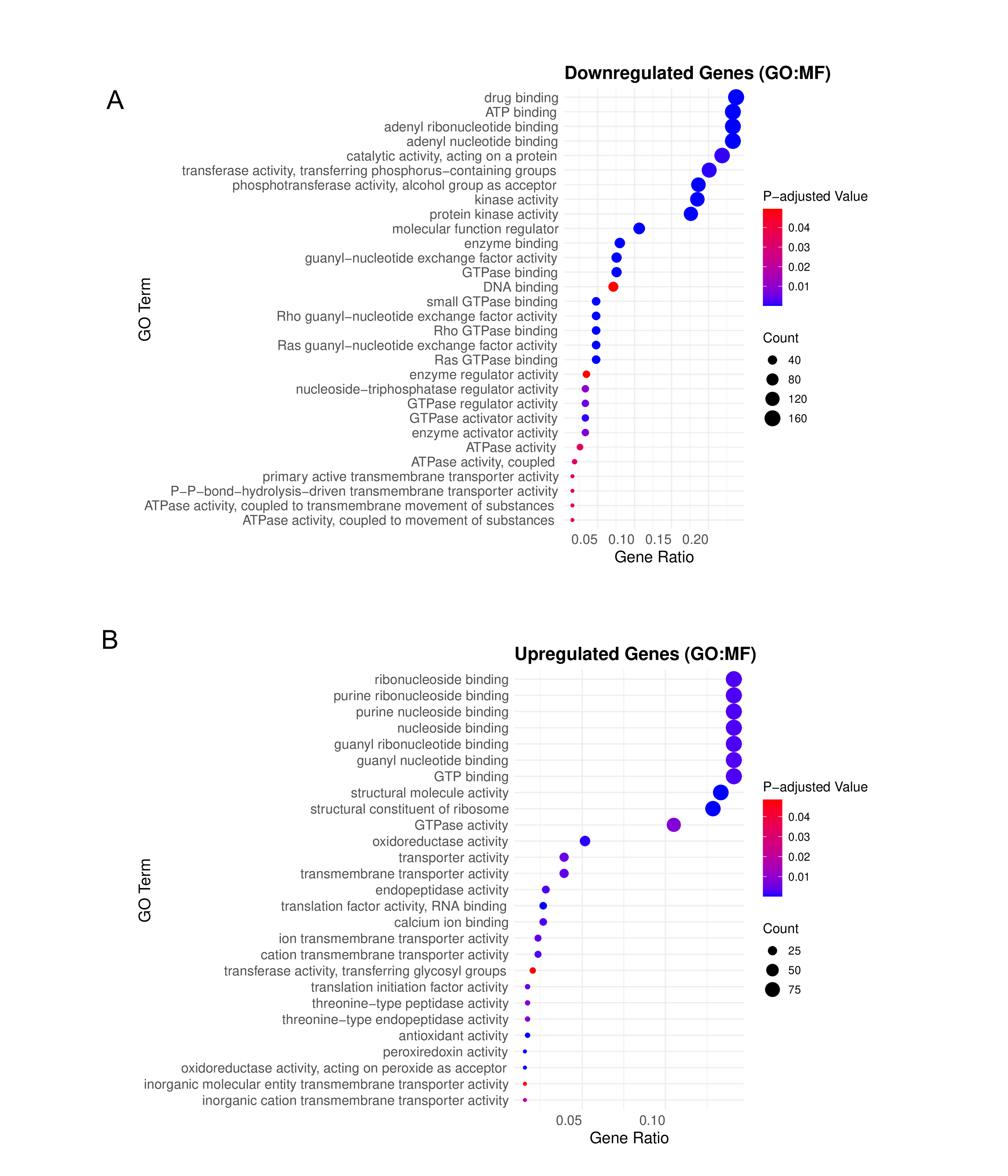

Supplement: S5 Fig — The result of GO enrichment analysis for downregulated (A) or upregulated (B) genes in EhracM gene silenced strain. All the differential genes with adjusted p-value (padj) < 0.05 were included in this analysis regardless of their foldchange compared to those of mock strain. GO terms that showed padj < 0.05 were selected in descending order of Gene Ratio (Ratio between the number of differentially expressed genes in each GO term and all differentially expressed genes that can be found in GO database) for each entry. Each dot size reflects the count size, whereas its color reflects the padj. The x-axis indicates Gene Ratio. (TIF) [file ppat.1012364.s005.tif]

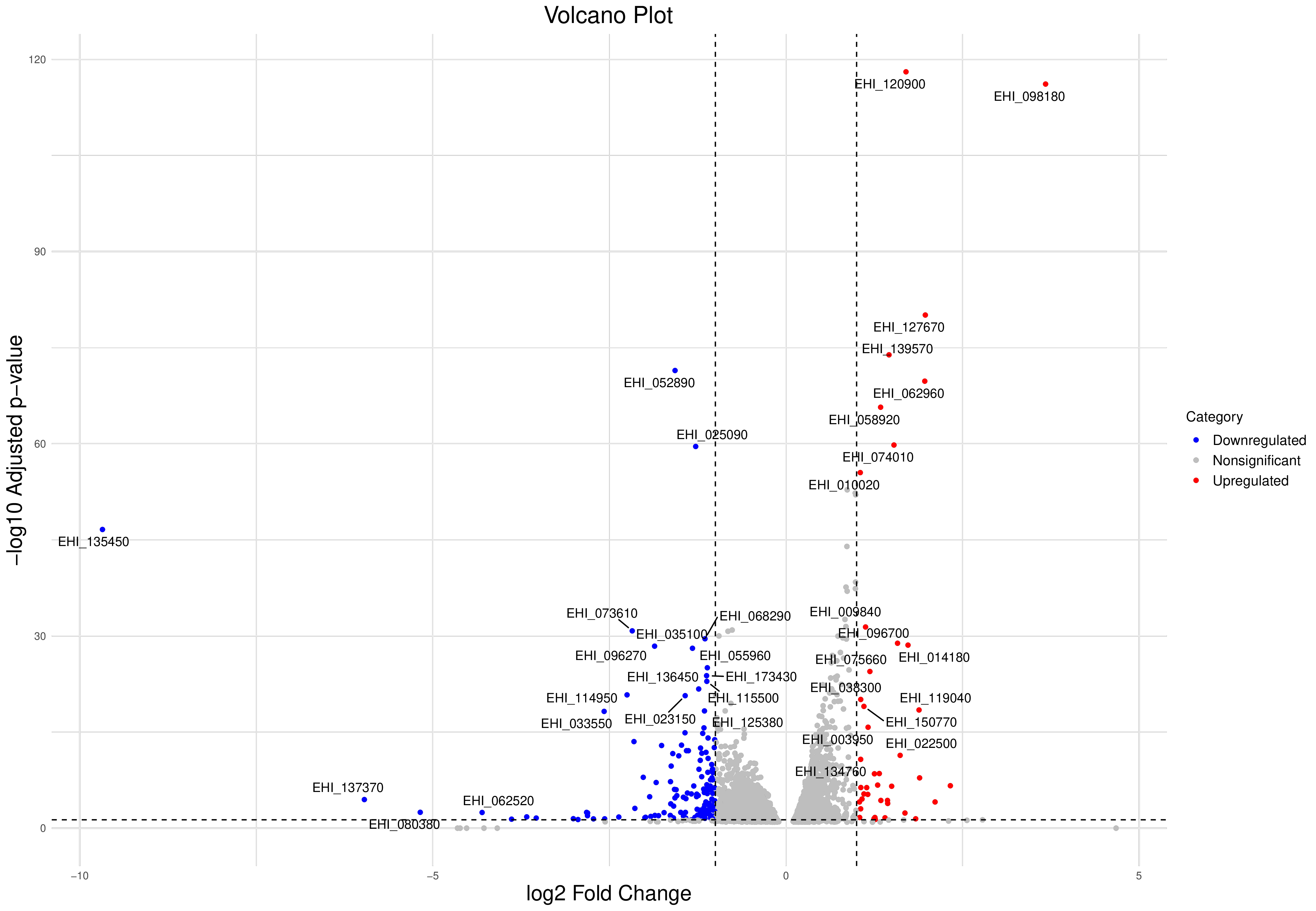

Supplement: S6 Fig — Volcano plots of the total RNA expression level of EhracM gene silenced strain vs psAP2 mock strain based on three biological replicates are shown. The x-axis reflects the folding change of total RNA amount in EhracM gene silenced strain compared with the psAP2 mock strain, whereas the y-axis shows the adjusted p-value (padj). Upregulated genes are shown in red, whereas downregulated genes are shown in blue. Others are shown in gray. (TIF) [file ppat.1012364.s006.tif]

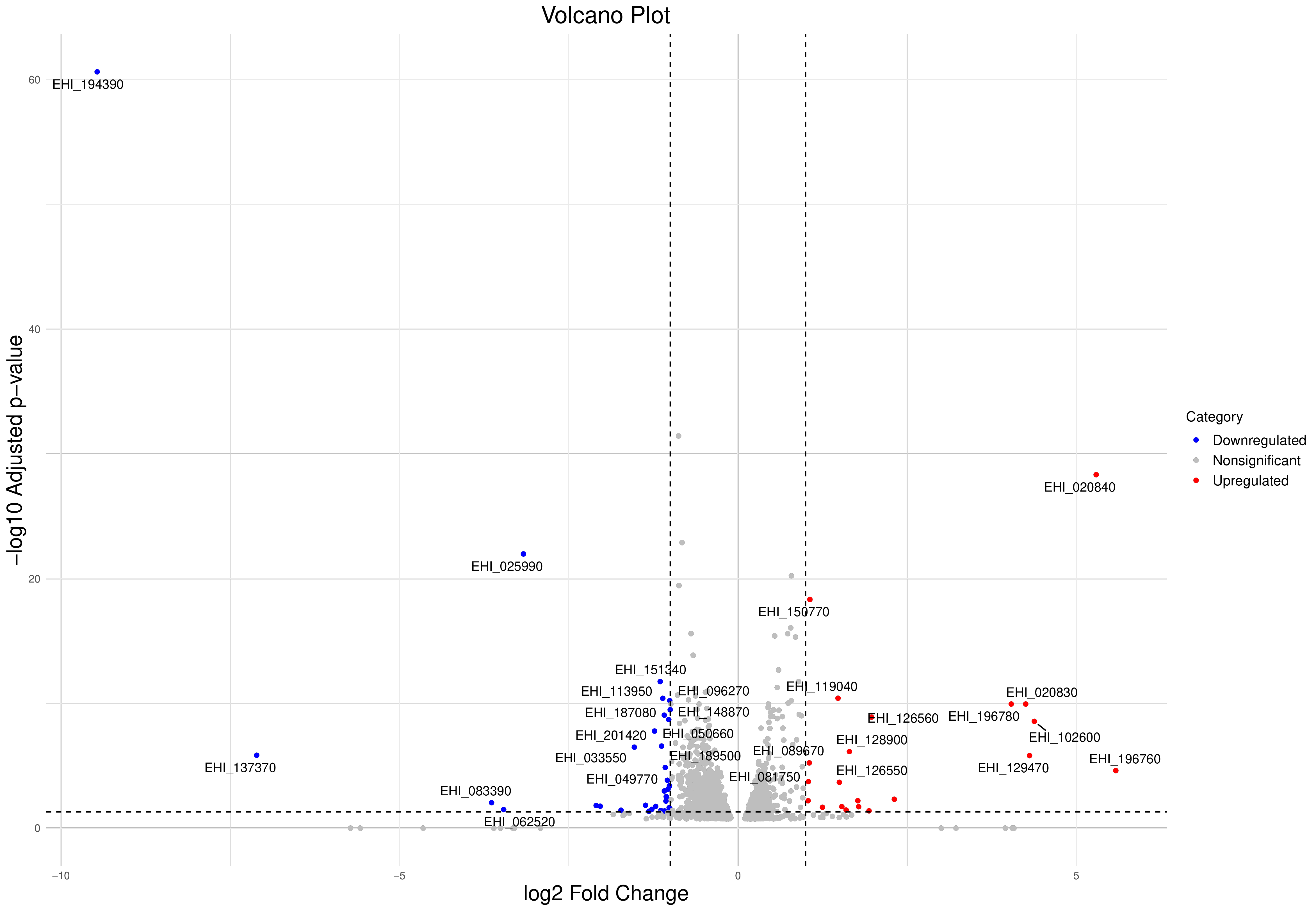

Supplement: S7 Fig — Volcano plots of the total RNA expression level of EhracJ gene silenced strain vs psAP2 mock strain based on three biological replicates are shown. The x-axis reflects the folding change of total RNA amount in EhracJ gene silenced strain compared with psAP2 mock strain, whereas the y-axis shows the adjusted p-value (padj). Upregulated genes are shown in red, whereas downregulated genes are shown in blue. Others are shown in gray. (TIF) [file ppat.1012364.s007.tif]

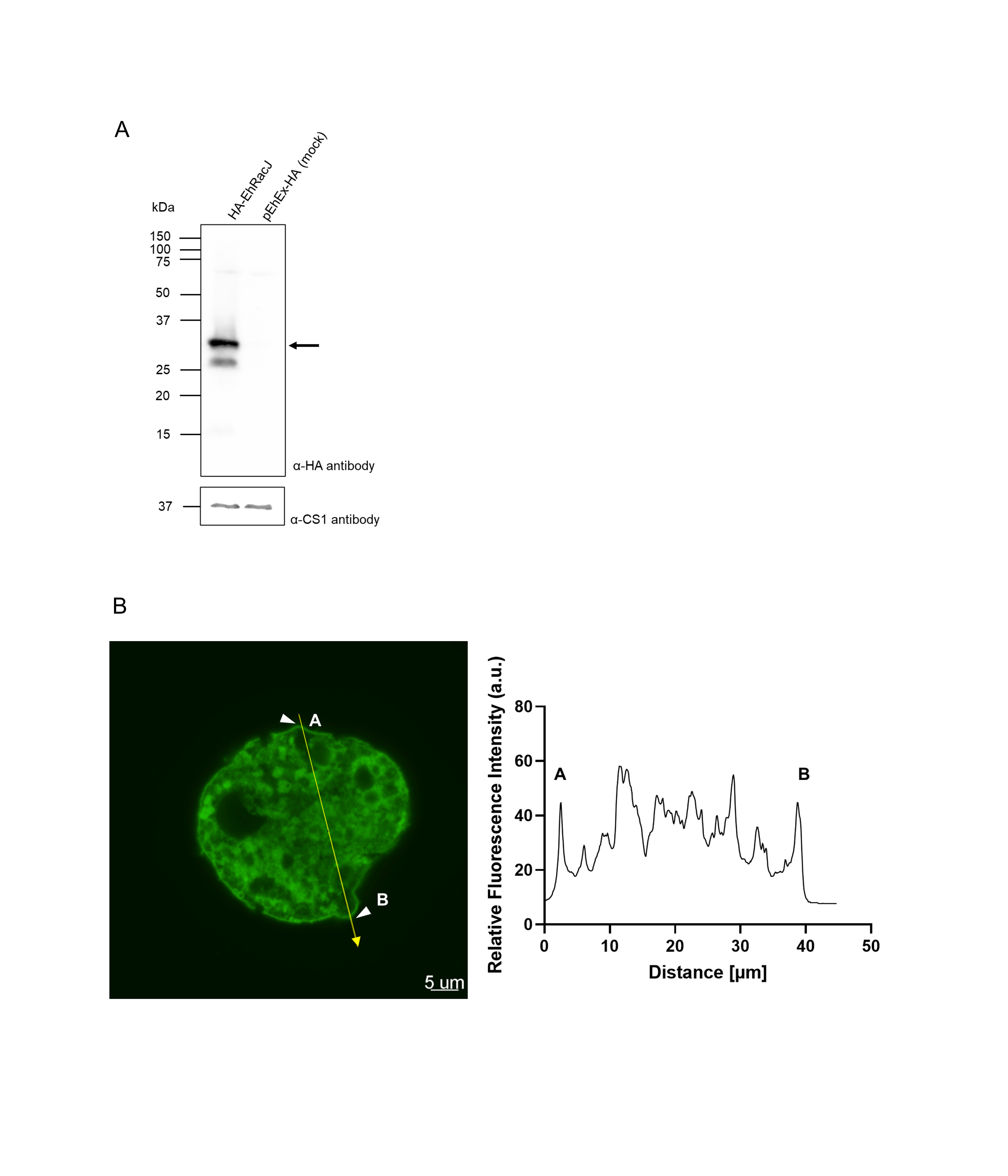

Supplement: S8 Fig — (A) Immunoblot detection of HA-EhRacJ in E. histolytica transformants. Approximately 30 μg of total lysates from mock-transfected control (pEhEx-HA) and HA-EhRacJ expressing transformants were subjected to SDS-PAGE and immunoblot analysis using anti-HA and anti-CS1 (loading control) polyclonal antibodies. The arrows indicate the approximate sizes of HA-EhRacJ. (B) The immunofluorescence image of HA-EhRacJ expressing trophozoite (left) and the line intensity plot along with the yellow arrow (right). (TIF) [file ppat.1012364.s008.tif]

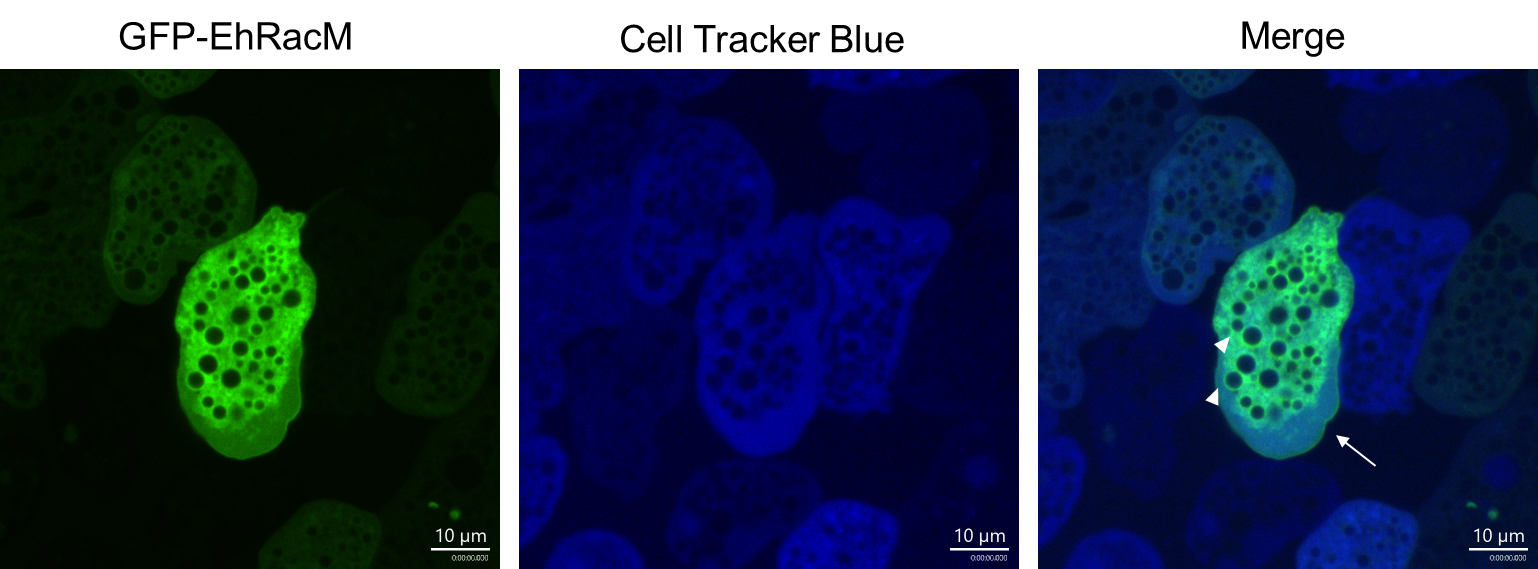

Supplement: S9 Fig — Representative image of live imaging of GFP-EhRacM (green) expressing trophozoite. GFP-EhRacM localizes mostly at the cytosol and sometimes plasma membrane (arrow) but enriches at the small vesicle surface (arrowheads). Cell Tracker Blue staining indicates the whole cytosol (blue). (TIF) [file ppat.1012364.s009.tif]

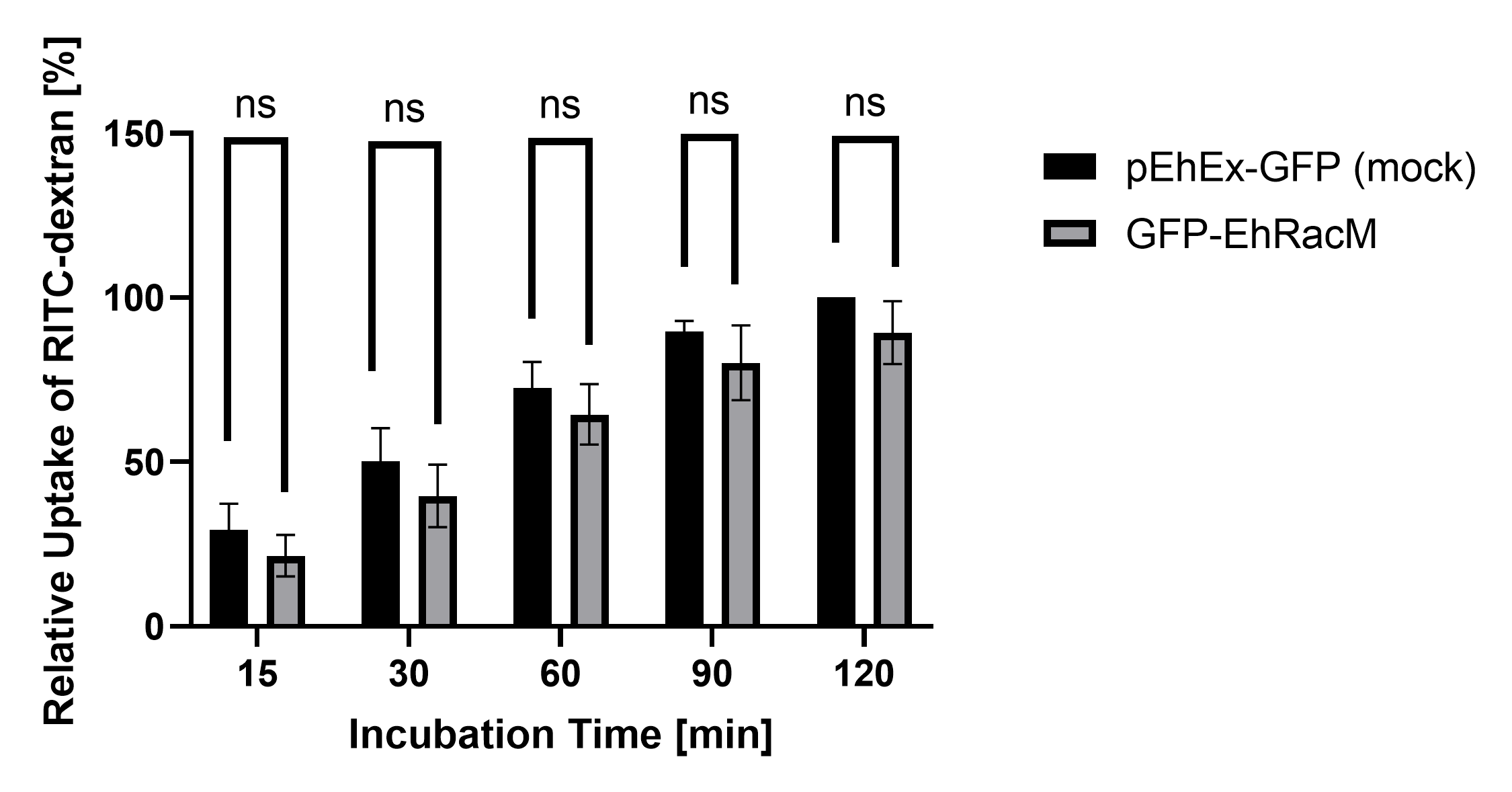

Supplement: S10 Fig — Trophozoites of GFP (mock) and GFP-EhRacM overexpressing strains were incubated in RITC dextran containing BIS medium to evaluate macropinocytosis. The fluorescence intensity of amoeba cells was measured by FACS as described in Material and Methods. Each value is standardized by the value of pEhEx-GFP mock control strain at 120 min. Statistical significance was examined with unpaired t-test (ns: not significant). Error bars indicate standard deviations of three biological replicates. (TIF) [file ppat.1012364.s010.tif]

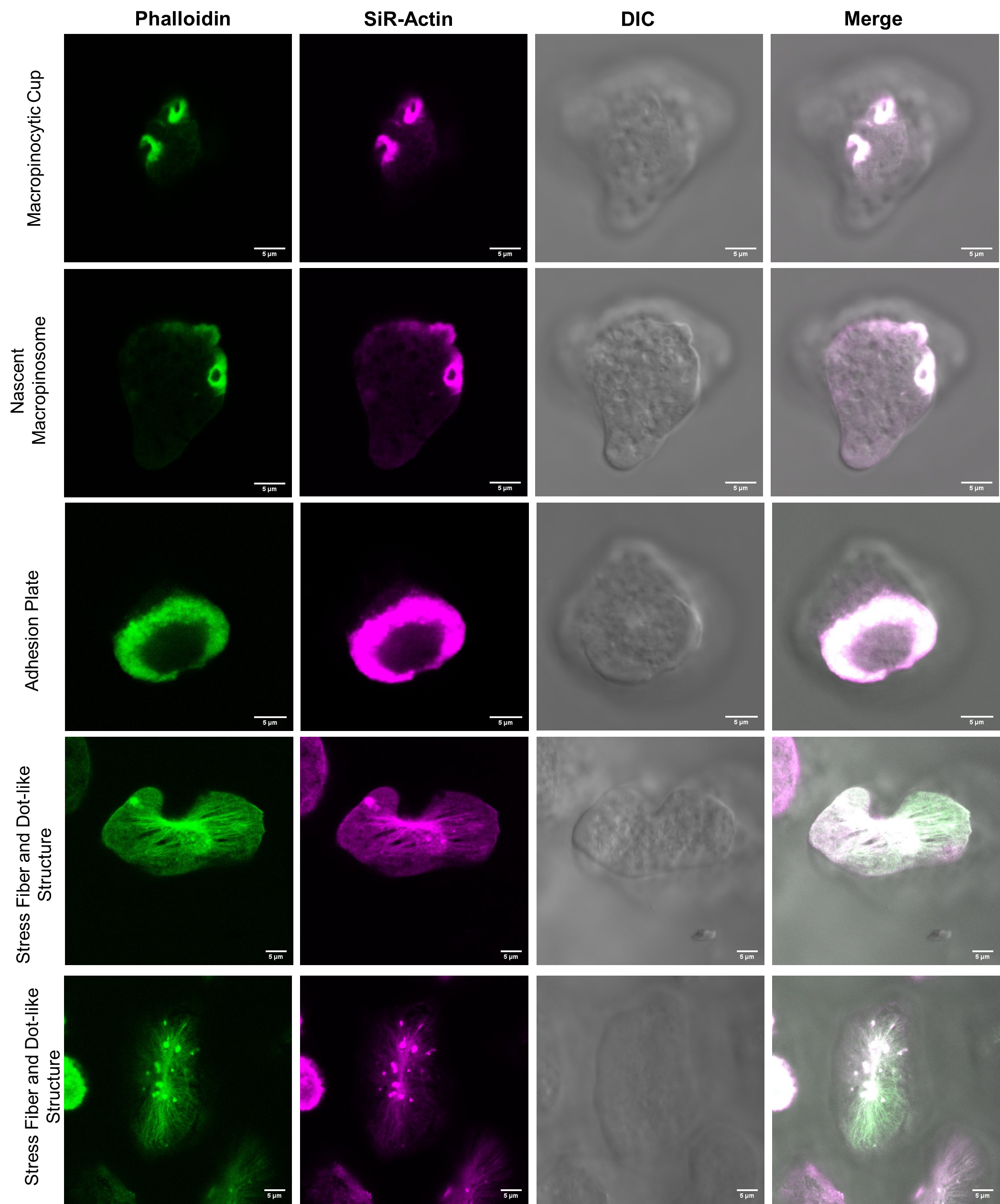

Supplement: S11 Fig — Trophozoites of E. histolytica HM-1:IMSS cl6 strain were double stained by phalloidin (green) and SiR-Actin (magenta). Each row shows representative actin-rich structures. (TIF) [file ppat.1012364.s011.tif]

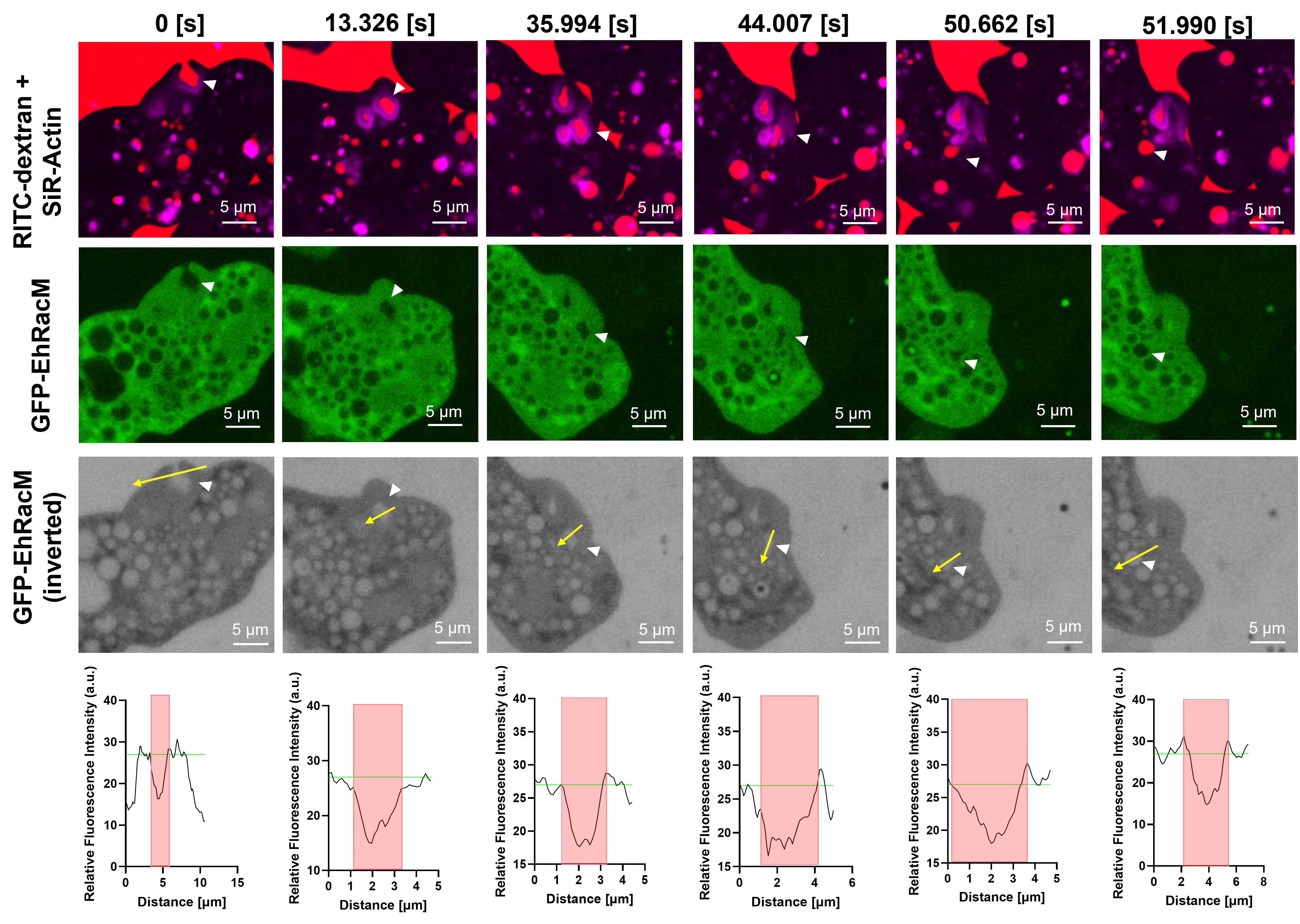

Supplement: S12 Fig — Montage of live imaging time series of a representative GFP-EhRacM expressing trophozoite in which macropinocytosis was monitored. F-actin was visualized using SiR-Actin (magenta), while GFP-EhRacM expressing trophozoites were shown in green (second row). The third-row panels indicate inverted grayscale signals of GFP-EhRacM. The trophozoites were incubated with RITC dextran (red). The white arrowheads indicate the site of macropinosome formation and the resultant macropinosome. The fourth-row panels show the GFP-EhRacM’s fluorescence intensity plot along with the trajectory of yellow arrows depicted in the corresponding third-row panels. Macropinosome areas are highlighted in red, and the mean GFP-EhRacM’s signal intensity at the initial macropinocytic cup (approximately 27) is shown in green. Bars, 5 μm. The F-actin envelope dissociation was captured at 50.662 [s]. (TIF) [file ppat.1012364.s012.tif]

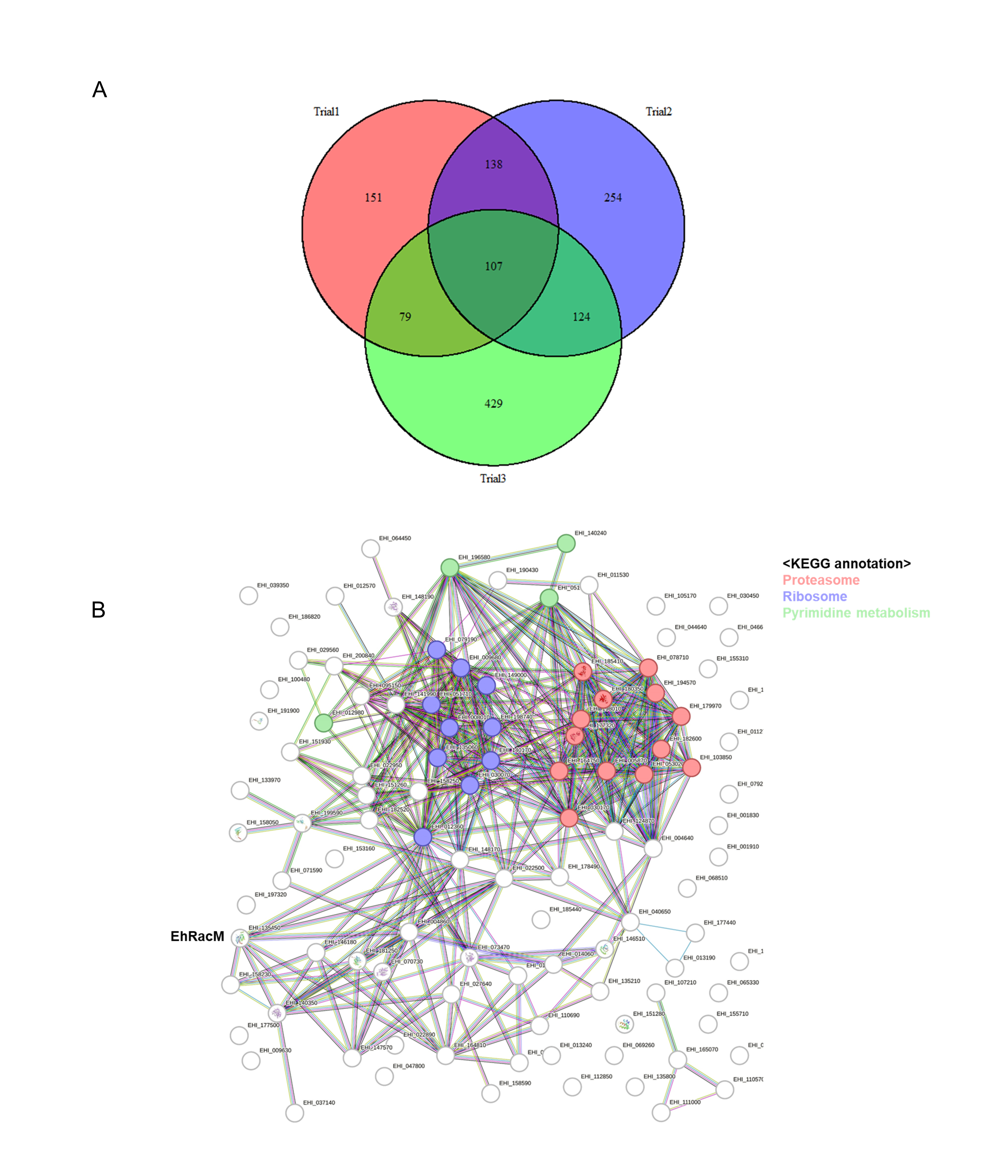

Supplement: S13 Fig — (A) Venn diagram of hit proteins in three independent co-IPs. Hits were defined as proteins whose QV was higher in HA-EhRacM than mock. Hits from the first co-IP are shown in red, hits from the second in blue, and hits from the third in green. 107 hits were identified in common in the three co-IPs, which are listed in S9 Table. (B) STRING protein-protein interaction network for the 107 proteins identified in common across the three co-IPs. Network nodes represent the proteins. Known interactions from curated databases are shown in sky blue, from experimentally determined are shown in magenta. Predicted interactions based on gene neighborhood are shown in green, based on gene fusions are in red, and gene co-occurrence is in blue. Moreover, textmining interactions are shown in yellow, co-expression proteins are tied in black, and protein homologies are shown in light blue. Proteins in the proteasome pathway (count in the network: 13 of 39, false discovery rate: 6.64e-12), the ribosome pathway (count in the network: 10 of 145, false discovery rate: 0.0016), and the pyrimidine metabolism (count in the network: 4 of 23, false discovery rate: 0.0116) in KEGG are shown as red, blue, and green nodes, respectively. (TIF) [file ppat.1012364.s013.tif]

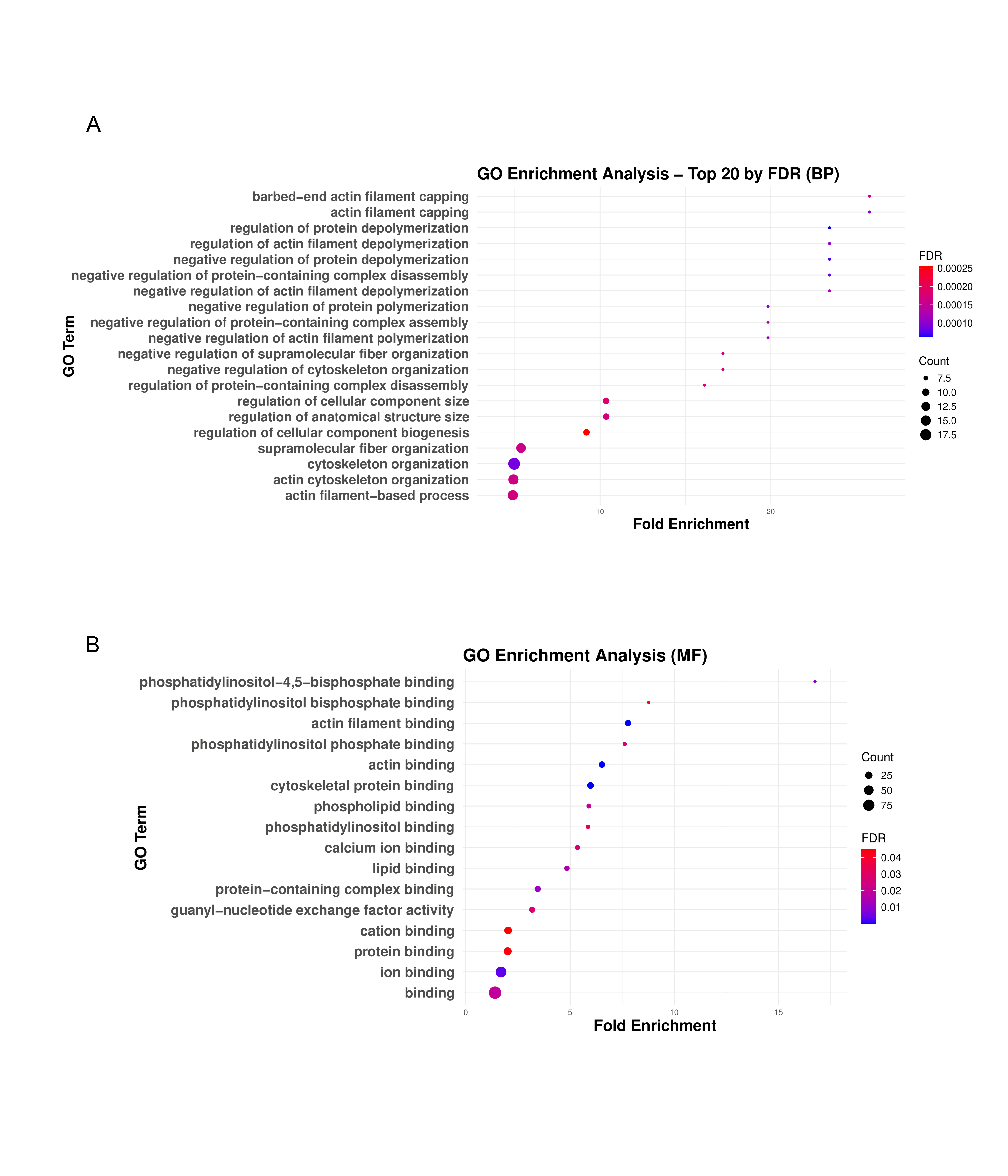

Supplement: S14 Fig — The results of PANTHER GO enrichment analysis on hit proteins from HA-EhRacJ co-IP. Proteins were classified by biological process (BP) (A) and molecular function (MF) (B). GO terms whose FDR-corrected p-value (FDR) are smaller than 0.05 are shown in descending order of FDR value for each entry (at most 20 terms). Each dot size reflects the count size, whereas its color reflects the FDR. The x-axis indicates fold change. (TIF) [file ppat.1012364.s014.tif]

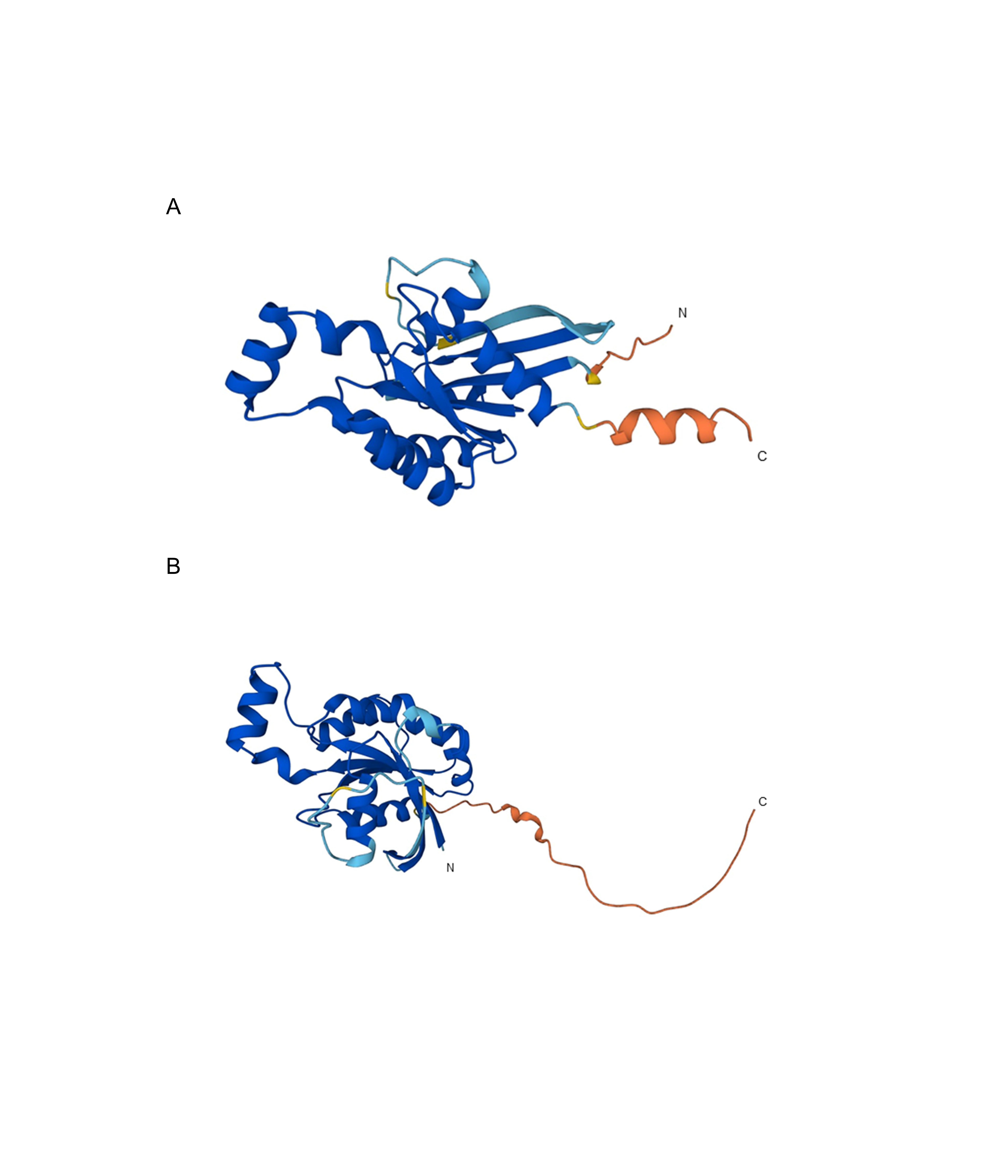

Supplement: S15 Fig — The predicted structures of EhRacM (C4M9S3) (A) and EhRacJ (C4LZV2) (B) were obtained from the AlphaFold Protein Structure Database (https://alphafold.ebi.ac.uk). N indicates the N-terminus, whereas C indicates the C-terminus of each protein. Each color represents the per-residue model confidence score (pLDDT), ranging from 0 to 100. Blue indicates a pLDDT greater than 90, light blue signifies scores between 70 and 90, yellow denotes scores between 50 and 70, and orange represents scores less than 50. (TIF) [file ppat.1012364.s015.tif]
