# Supplementary material for: EhRacM differentially regulates macropinocytosis and motility in the enteric protozoan parasite Entamoeba histolytica
Source: PLoS Pathog. 2024 Nov 13;20(11):e1012364. doi: 10.1371/journal.ppat.1012364 (PMC11560011; doi:10.1371/journal.ppat.1012364)
Supplement: Attachment — Submitted filename: Point to reviewers.pdf [file ppat.1012364.s031.pdf]

Point-to-point responses to reviewers' comments

We sincerely appreciate all the constructive comments from the referees. Please see below our point-to-point responses to all the points raised.

Referee #1

Major Issues: Key Experiments Required for Acceptance

1. "Potentially, one of the most important findings is that EhRacM accumulates on macropinosomes after they are formed (i.e., EhRacM does not accumulate on forming cups). The data supporting this finding are first shown in Figure 5 and form the basis for many of the next experiments (Figs. 6-8). Although the accumulation of EhRacM around one of the macropinosomes (white arrowhead) is evident and supported by the intensity measurements, there are other RITC-filled macropinosomes in the image that don't seem to have an accumulation of EhRacM. For example, there is one just below the endosome labeled with the arrowhead that doesn't seem to be enriched in EhRacM. This reviewer understands that there are different planes of focus, but it raises the question about whether the EhRacM-enriched endosome is an exception. It would be helpful to quantify the % of total macropinosomes that become enriched with EhRacM."

Our response: We appreciate your insightful comment regarding the accumulation of EhRacM on macropinosomes. As you suggested, we have quantified the percentage of total macropinosomes that become enriched with EhRacM.

To address this, we conducted an experiment where GFP-EhRacM overexpressing *E. histolytica* trophozoites were incubated with the BIS medium containing RITC dextran for 15 minutes (pulse), transferred to fresh medium without RITC dextran, and images were taken at 0, 15, 30, 60, and 120 minutes after "pulse," using a spinning disk confocal microscope. We analyzed the images, using FIJI ImageJ software, to measure the signal in two channels: RITC dextran-filled macropinosomes (red) and GFP-EhRacM (green). We used the red channel to identify macropinosomes, while the green channel was used to monitor the accumulation of EhRacM. To evaluate EhRacM association to macropinosomes without bias, we first estimated the background fluorescence level of the cytoplasm by measuring the average fluorescence intensity in the green channel of three randomly selected cytosolic regions (a circle of 2 µm diameter) and set this background level as the threshold of the cytoplasmic background signal. We then defined GFP-EhRacM-positive pixels when the detected green signal in each pixel was above the threshold. A macropinosome containing an area with the red fluorescent signal was considered to be 'GFP-EhRacM-positive' when the area outside of the macropinosome facing the cytoplasm is continuously surrounded along > 50% of the perimeter of the macropinosome membrane. We analyzed 45, 49, 37, 50, and 23 macropinosomes at 0, 15, 30, 60, and 120

minutes, respectively.

Our analysis nicely showed a time-dependent increase in the percentage of EhRacM-associated macropinosomes (62.2%, 67.3%, 91.9%, 92.0%, and 95.7% at time points indicated above, respectively), supporting our hypothesis that EhRacM accumulates on matured macropinosomes. We must admit that this analytical method is subjective. Since the Editor stated that this suggested experiment is not necessary for the revision of the manuscript, we opted not to include these results in the revised manuscript.

Minor Issues: Editorial and Data Presentation Modifications

1. "In the introduction (Line 62), it sounds like viruses, bacteria and prions also exhibit their own micropinocytosis. This reviewer knows it is not what the authors mean (they mean that some of these pathogens are taken up into host cells by host cell endocytosis, but it is not clear as written."

Our response: We agree with your suggestion. To clarify, we have revised the sentence to: "the role and molecular mechanisms of macropinocytosis in the pathogenesis of eukaryotic pathogens remain to be elucidated." (Page 4, Lines 61–62)

2. "In Line 123, the authors should include the gene name (rho1B) in addition to XP_651936. In this way, readers won't need to backtrack to figure out what rac/rho could not be silenced."

Our response: We have updated the text to include the gene name as requested. The revised sentence now reads: "XP_651936 (EHI_013650 (*EhracR2*) or EHI_068240 (*EhracR1*))." (Page 7, Line 124)

Referee #2

Minor Issues: Editorial and Data Presentation Modifications

1. "The author have introduced EhRacM as Rho like molecule but the sequence alignment with other known Rho like EhEhRho1 and host Rho proteins should be done to explain the relatedness and status of various domains and motifs in EhRacM. Incorporation of Phylogenetic analysis will be better to explain if it belongs to Rho class of GTPase."

Our response: We have added in the revision multiple sequence alignments of HsRhoA (NP_001300870.1), HsRac1 (NP_008839.2), HsCdc42 (NP_001034891.1), EhRho1B (EHI_029020), EhRacA2 (EHI_197840), EhRacD1 (EHI_012240), EhRacG (EHI_129750), and EhRacM (EHI_135450) (Supplementary Figure 1). This analysis highlights the conservation of key Rho motifs in EhRacM, including four G-box regions, Rho insert regions, and effector regions (Wu *et al.*, ***Journal of Biological Chemistry***, 1997; Freeman *et al.*, ***Journal of Biological Chemistry***, 1996). The phylogenetic analysis suggested by this reviewer was

already conducted and reported by Hon *et al.* (2010), which we have referenced from the book chapter *Dissecting the Actin Cytoskeleton of Entamoeba histolytica from a Genomic Perspective* in *Anaerobic Parasitic Protozoa: Genomics and Molecular Biology* (Page 98).

2. "Authors have used Verapamil to stain actin by SiR probe (line 818) in fixed trophozoites. However, verapamil is used for achieving higher labelling intensities in live cells during real time imaging. Can author explain why Verapamil was used for fixed cells?"

Our response: We acknowledge the reviewer's observation. Verapamil, an L-type calcium channel blocker, has been used to inhibit efflux pumps, and thus enhance staining with SiR-actin in live cells (G. Lukinavičius *et al.*, ***Nature Methods***, 2014). Thus, the use of verapamil for fixed trophozoites was a mistake due to our misunderstanding. However, we believe the use of verapamil did not affect our data and their implications.

3. "According to figure 3A, the results indicate that EhRacM is involved in linear motility of trophozoites. However, the results are performed in normal conditions and random motility event is selected. If the motility assay can be performed in presence of some chemotactic agent like TGF-beta or cAMP gradient, then the comment on linear motility can be more affirmative."

Our response: We agree with this suggestion. Chemotaxis of *E. histolytica* was indeed previously reported (Franco-Barraza *et al.*, ***Cell Motility and the Cytoskeleton***, 2006; Blazquez *et al.*, ***Infection and Immunity***, 2006). However, despite our attempts to replicate these methods, we encountered significant challenges that prevented us from obtaining reliable results. Additionally, we currently lack access to the necessary live imaging platform, such as an anaerobic constant flow chamber under a fluorescent microscope, which would be required to properly visualize and quantify chemotactic responses. Once we overcome these technical challenges, we will definitely explore this convincing experiment in the future, which would provide more definitive insights into the role of EhRacM in motility or even in macropinocytosis.

4. ***"Authors state that EhRacM is associated with macropinocytic vesicles for a long time and help in maturation (Fig 6). As this protein is also involved in phagocytosis and trogocytosis. Has it been reported in phagosome proteomes of E. histolytica reported by various authors?"***

Our response: Yes, EhRacM was detected in the proteome of phagosomes isolated using the magnetic beads, as reviewed and summarized (Watanabe *et al.*, ***Genes***, 2023). It is of note that EhRacM was only detected from the phagosomes isolated with serum-coated and non-coated magnetic beads (Watanabe *et al.*, ***Cellular microbiology***, 2020; Nakada-Tsukui *et al.*, Frontiers

in cellular and infection microbiology, 2022; Marion et al., ***Cellular microbiology***, 2005), but not from the phagosomes isolated by the floatation method using latex beads (Okada *et al.*, ***Eukaryot Cell***. 2005; Okada *et al.*, ***Molecular and Biochemical Parasitology***, 2006; Furukawa *et al.*, ***Infection and Immunity***, 2013). The demonstration of EhRacM from phagosomes under certain conditions and time points indicates a rather transient or indirect interaction of EhRacM with phagosomes. However, our previous indirect immunofluorescence assay results did not clearly show the localization of EhRacM on phagosomes (data not shown). Therefore, we would rather not discuss this point in the manuscript.

5. "What is the status of expression of this gene in virulent versus avirulent strains? Does virulent pathogen show decreased expression of this gene? Does treatment of trophozoites with Rac-GTPase inhibitors or constitutive expression of catalytically nonfunctional EhRacM mimics same effect as gene silenced one? Perform at least one of the experiments to validate gene silencing function."

Our response: According to RNA-seq data from Weber *et al.* (***Scientific Reports***, 2016), *EhracM* expression was highest in virulent parasites (HM1-IMSS) recently isolated from liver abscesses of infected hamsters, compared to attenuated HM1-IMSS parasites that had lost the ability to form liver abscesses after long-term culture, HM1-IMSS parasites cultured for a short time (less than three months) in vitro, and HM1-IMSS parasites exposed to explants of the human colon (VirColon, (Bansal *et al.*, ***PLoS Neglected Tropical Diseases***, 2009)). Based on our own transcriptomic analysis of animal-passaged highly virulent strain and attenuated avirulent strain, no apparent changes in the steady state mRNA level were observed (Saito-Nakano *et al.*, ***Frontiers in Cellular and Infection Microbiology***, 2021). Similar observations were also reported (Meyer *et al.*, ***PLoS Pathogens***, 2016)". Thus, we have to tentatively conclude the *EhRacM* expression levels vary under different laboratory culture conditions and the genetic background of the strains, and there is no clear causal association with virulence.

6. "Schematic representation of the model put forward by the authors in discussion part will be better for understanding by the readers."

Our response: For a better understanding, we have added Figure 10 to illustrate the working model of EhRacM in macropinocytosis.
